# Self-Compassion Scale: IRT Psychometric Analysis, Validation, and Factor Structure – Slovak Translation

**DOI:** 10.5334/pb.398

**Published:** 2018-01-04

**Authors:** Júlia Halamová, Martin Kanovský, Monika Pacúchová

**Affiliations:** 1 Institute of Applied Psychology, Faculty of Social and Economic Sciences, Comenius University in Bratislava, Mlynské luhy 4, 821 05 Bratislava, SK; 2 Institute of Social Anthropology, Faculty of Social and Economic Sciences, Comenius University in Bratislava, Bratislava, SK

**Keywords:** self-compassion, self-compassionate responding, self-uncompassionate responding, IRT factor analysis, two-tier model

## Abstract

The present study verifies the psychometric properties of the Slovak version of the Self-Compassion Scale through item response theory, factor-analysis, validity analyses and norm development. The surveyed sample consisted of 1,181 participants (34% men and 66% women) with a mean age of 30.30 years (SD = 12.40). Two general factors (Self-compassionate responding and Self-uncompassionate responding) were identified, whereas there was no support for a single general factor of the scale and six subscales. The results of the factor analysis were supported by an independent sample of 676 participants. Therefore, the use of total score for the whole scale would be inappropriate. In Slovak language the Self-Compassion Scale should be used in the form of two general subscales (Self-compassionate responding and Self-uncompassionate responding). In line with our theoretical assumptions, we obtained relatively high Spearman’s correlation coefficients between the Self-Compassion Scale and related external variables, demonstrating construct validity for the scale. To sum up, the Slovak translation of The Self-Compassion Scale is a reliable and valid instrument that measures Self-compassionate responding and Self-uncompassionate responding.

Over the past fifteen years, there has been a raise in scientific and clinical interest in self-compassion. Self-compassion, which is compassion directed inwards, involves treating the self with care and concern when faced with the experience of suffering, rather than with a sense of self-criticism (Neff & Dahm, 2015). Contrary to widely held popular beliefs that self-criticism may serve a positive role in motivation and success (Baker & McNulty, 2011; Breines & Chen, 2005), a large and growing body of research has found that self-compassion also has superior psychological, physical and social benefits that support a happy, healthy and successful life (Dunne, Sheffield, & Chilcot, 2016; Gilbert, Clark, Hempel, Miles, & Irons, 2004; Neff, 2004). For this reason, there has been a growing tendency to translate psychometrically validated tools to measure self-compassion in various languages worldwide. The Self-compassion scale (Neff, 2003) was translated into Chinese (Chen, Yan, & Zhou, 2011), Czech (Benda & Reichová, 2016), Dutch (Neff & Vonk, 2009), French (Kotsou & Leys, 2016), German (Hupfield & Ruffieux, 2011), Greek (Mantzios, Wilson, & Giannou, 2015), Hungarian (Tóth-Király, Bőthe, & Orosz, 2016), Iranian (Azizi, Mohammadkhani, Lotfi, & Bahramkhani, 2013), Italian (Petrocchi, Ottaviani, & Couyoumdjian, 2014), Japanese (Arimitsu, 2014), Korean (Lee & Lee, 2010), Norwegian (Dundas, Svendsen, Wiker, Granli, & Schanche, 2016), Portugese (Castilho, & Pinto-Gouveia, 2011), Spanish (Garcia-Campayo, Navarro-Gil, Andrés, Montero-Marin, López-Artal, & Demarzo, 2014), Thai (Pisitsungkagarn, Taephant, & Attasaranya, 2014), and Turkish language (Deniz, Kesici, & Sumer, 2008).

The Self-Compassion Scale (SCS; Neff, 2003) is the most commonly used scale to measure self-compassion. Consisting of 26 items, the SCS measures self-compassion through three interrelated dichotomies (six subscales): Self-Kindness (SK) versus Self-Judgment (SJ), Common Humanity (CH) versus Isolation (IS), and Mindfulness (MI) versus Over-identification (OI). Since its development, the SCS has been found to be a powerful predictor of meaningful outcomes. For example, self-compassion is associated with social solidarity and lower levels of rumination, depression and anxiety (Neff, Kirkpatrick, & Rude, 2007; Raes, 2011). Furthermore, people with greater self-compassion are known to be less emotionally affected by critical feedback, have less fear of failure and persist longer on challenging tasks relative to those low on self-compassion, indicating that self-compassion is a helpful source of motivation and resilience (Leary et al., 2007; Neff, Hsieh, & Dejitterat, 2005).

The reliability of the SCS scale has been investigated by various authors, beginning with the original scale validation study of Neff (2003). Neff (2003) found high internal consistency for the overall scale (α = 0.92), and also for each subscale (α = 0.75–0.81). Likewise, Neff found a high test-retest reliability over a three-week period of time for both the whole scale (*r* = 0.93), as well as for the individual subscales – Self-Kindness (*r* = 0.88), Self-Judgment (*r* = 0.88), Common humanity (*r* = 0.80), Isolation (*r* = 0.85), Mindfulness (*r* = 0.85), and Over- identification (*r* = 0.88). In accordance with Neff (2003), high internal consistency of the scale has also been reported by Van Dam et al. (2011) (full scale α = 0.92; subscales ranging between 0.72 and 0.83). Turkish and Czech versions of the SCS have similarly demonstrated good internal consistency, and good test-retest reliability, indicating appropriate reliability of translated versions of the scale (Benda & Reichova, 2016; Deniz et al, 2008).

In addition to scale reliability, authors have demonstrated that the SCS has evidence of convergent and discriminant validity with associated constructs. For example, Neff (2003) reported a significant negative correlation between self-compassion and the subscale of self-criticism in the Depression Experience Questionnaire (*r* = –0.65, *p* < 0.01), and a significant positive correlation with the scale of social connection (*r* = 0.41, *p* < 0.01. More recently, Benda and Reichová (2016), confirmed the convergent validity on the basis of a positive correlation of the scale with questionnaires of mindfulness (*r* = 0.62, *p* < 0.01), self-acceptance (*r* = 0.58, *p* < 0.01) self-esteem (*r* = 0.73, *p* < 0.01) and discriminant validity of the SCS scales verified by the scale measuring the intensity of non-clinical narcissism (*r* = –0.31, *p* < 0.01).

While the reliability and validity of the scale has been reported by a number of independent research groups (e.g. Brenda & Reichová, 2016; Deniz et al., 2008; Neff, 2003; Van Dam et. al., 2011), the factor structure of the SCS has been more contentious. In the original study, Neff (2003) used confirmatory factor analysis (CFA) to verify a three-factor model of self-compassion including the subscales of Self-Kindness, Common humanity and Mindfulness, however the model resulted in inadequate fit to the data (CFI < 0.90). Subsequently, based on modification indices, Neff (2003) revised her model to separate positively and negatively worded items to generate the now widely used six-factor model of the SCS. While this final model was associated with good factor loadings, the inter-correlations between the six subscales were high (–0.46 to 0.91). Consequently, to explain these high factor inter-correlations, a higher order general ‘self-compassion’ factor was included in the CFA model.

Unfortunately, some recent factor analytic studies have failed to confirm Neff’s (2003) factor structure of the SCS described above. For example, in their Czech translation validation study, Benda and Reichová (2016) found evidence of six problematic items (items 3, 9, 15, 21, 22 and 23) that substantially hindered model fit. Likewise, in the Italian translation some problematic items emerged (items 15 and 23), whereby the authors recommended their removal from the scale (Petrocchi et al., 2014).

Benda and Reichová (2016) in their Czech version of the scale also did not succeed in confirming the models mentioned above, nor did they find evidence for the three-factor model, while once again it was demonstrated that the scale had high intercorrelations between subscales. After removing six problematic items from the scale, they confirmed a six-factor structure with one common factor of a higher order – self-compassion. Also, Benda and Reichová (2016) reported high intercorrelations between the subscales, which diminished after the removal of the mentioned problematic items. Similarly, the Italian version of the SCS confirmed a six-factor model and did not show one higher-order factor for self-compassion. Items 15 and 23 were loaded on several factors, so they recommended omitting them. The authors tested the single-factor and two-factor model but neither of them showed to be suitable (Petrocchi et al., 2014). Together, these findings demonstrate that the factor structure and item functioning in the SCS is still somewhat unclear.

Some recent studies from a range of independent research groups have also demonstrated the feasibility of a two general factor solution separating positively formulated items (Self-Kindness, Mindfulness and Common Humanity) from negatively formulated items of self-compassion (Self-Judgement, Over-identification and Isolation) (Brenner, Heath, Vogel, & Credé, 2017; Brown, Bryant, Brown, Bei & Judd, 2016; Costa, Marôco, Pinto-Gouveia, Ferreira, & Castilho, 2016; López, Sanderman, Smink, Zhang, van Sonderen, Ranchor, & Schroevers, 2015; Muris & Petrocchi, 2016). The breadth of research supporting this two factor solution is quite compelling, demonstrating the potential need to delineate positive and negative self-compassion.

## Aim of the Study

The central aim of this study was to investigate the psychometric properties and factor structure of the Slovak translation of The Self-Compassion Scale (SCS) using item response theory (IRT). In particular, we were interested in verifying the original factor structure reported by Neff (2003) that included six subscales, the factor structure that included one general factor ‘self-compassion’ with six subscales (the bifactor model, where each item loaded on its specific factor and on the general factor, see Neff, 2003), and also the two-tier model (Bonifay, 2016; Cai, 2010; Cai, 2016) where each item loaded on its specific factor and on the one of two general factors representing positive and negative part of self-compassion. The bifactor model (Reise, 2012; Reise et al., 2010) allows separating variance accounted for by a single general factor from the variance accounted for by specific factors: if there is a single strong general construct (self-compassion) over and above the six subscales, the bifactor model should have better fit than the six-factor correlated model. On the other hand, if there are two general constructs over and above the six subscales, the two-tier model should have better fit than the bifactor model. Since these models are nested (the two-tier model is the most general of these models), we can compare them directly by means of the likelihood-ratio tests. We were also interested in considering the factor structure reported by other authors (e.g. Brenner et al., 2017; Brown et al., 2016; Costa et al., 2016; López et al., 2015; Muris & Petrocchi, 2016) that delineates positively formulated items and negatively formulated items of self-compassion through two general factors. There are two indirect ways to detect this: (1) the inspection of the magnitude of correlations among six latent factors in the six-factor model: if the correlations within the positive and negative subdimensions are stronger than the correlations between them, it is indicated that positive and negative subdimensions form two distinct groups; (2) the inspection of factor loadings of the general factor in the bifactor model: if factor loadings of positive and negative items significantly differ, this indicates that a single general factor does not explain sufficient amount of variance and that two general factors are present. Finally, we also tested the reliability, validity of this scale, its scalability by means of non-parametric IRT analysis (van der Ark, 2012) and created norms for the scale, to enable use of the Slovak translation of the SCS in future research.

## Research Methods

### Measuring instruments

**The Self-Compassion Scale** (SCS; Neff, 2003) measures six aspects of self-compassion in situations of a perceived difficult time. The scale includes 26 items rated on a 5-point Likert-type Scale of frequency (1 = almost never; 5 = almost always). The subscale Self-Kindness (SK) represents the ability of taking care of oneself and being warm towards oneself when encountering failure situations. Common Humanity (CH) reflects the personal understanding that suffering is part of the shared human experience. Mindfulness (MI) is a non-judgmental state of mind in which individuals observe their thoughts and feelings as they are, without over-identification or without trying to suppress or deny them. They are seen as either “negative” or “positive”. The scale measures the degree to which individuals display self-kindness against self-judgment, common humanity versus isolation, and mindfulness versus over-identification. The Over-identification (OI), Isolation (IS) and Self-Judgment (SJ) subscales are therefore scored negatively. The total score of the scale is calculated by the average of individual subscales, while a negatively scored item must be transformed. In Slovak language, we did back translation of the scale and the discrepancies were discussed and decided by consensus. The items of the English and Slovak versions of SCS are in Appendix 1.

**The Forms of Self-criticism/Attacking & Self-Reassuring Scale** (FSCRS; Gilbert et al., 2004) is a 22-item instrument which was developed to determine the level self-criticism and the ability of self-reassurance. On a 5-point Likert Scale, participants rated the extent to which various statements are true about themselves (1 = not at all like me; 5 = extremely like me). The questionnaire consists of 22 items, which measure how a person feels and thinks in severe, adverse life situations. The scale comprises three scales: Inadequate Self (IS) which focuses on feelings of personal inadequacy, Hated Self (HS) measuring the desire to hurt or punish oneself, and Reassured Self (RS) which is an ability of self-affirmation.

**The Levels of Self-Criticism Scale** (LOSC; Thompson & Zuroff, 2004) was developed to measure two dysfunctional forms of negative self-evaluation: Comparative Self-Criticism (CSC) and Internalized Self-Criticism (ISC). The scale contains 22 items and measures Comparative Self-Criticism (CSC), which is defined as the negative view of the one’s self, acquired by comparison with other people. Internalized Self-Criticism (ISC) is the negative view of the self, which is formed by comparing oneself with one’s own personal standards and objectives. It consists of 12 items measuring the Comparative Self-Criticism subscale and 10 items measuring the Internalized Self-Criticism subscale. Participants answered the items on a Likert scale from 1 = not at all to 7 =very well.

**The Self-Compassion and Self-Criticism Scales** (SCCS; Falconer, King, & Brewin, 2015) measures two dimensions: *Self-criticism* (SCR) and *Self-compassion* (SCO). It consists of five self-threatening scenarios describing various situations that have the potential to induce people to varying degrees of self-criticism or self-compassion. On a 7-point scale (1-not at all 7- to highly), respondents indicated the extent to which they would respond reassuringly, soothingly, contemptuously, compassionately, critically and harshly to these situations.

### The Research Sample

The research sample included 1,181 participants of whom 402 were males (34%) and 779 females (66%). The mean age was 30.30 years (SD = 12.40), and ranged from 18 to 82 years. 667 male and female respondents were single, and 514 were in relationship. With regard to education, 152 respondents (13%) had completed primary education, 572 (48%) had completed secondary school education and 457 (39%) had a university degree.

An independent research sample was used only for validating our factor analysis. This sample included 676 participants out of which 15 % were male and 85% were female. Their mean age was 29.90 years (SD = 11.21).

### Data Collection

Data was collected gradually over two and half years within a research grant focused on self-criticism and self-compassion. Data was obtained by convenience sampling; questionnaires were distributed on paper and also in a digital form via social networks. The authors declare that there are no conflicts of interest and confirm complying with APA ethical principles in the treatment of individuals participating in the research. The research has been carried out in accordance with The Code of Ethics of the corresponding University.

### Data Analysis

For data recording, we used the program SPSS Statistics-20 and for statistical processing the software R (version 3. 1. 3, R Core Team, 2015) packages psych (Revelle, 2015), mirt (Chalmers, 2012), and mokken (Van der Ark, 2012) were used. The procedure was as follows: (1) Descriptive analysis: standard distributive properties of items, as well as testing univariate normal distributions of items and multivariate normal distribution of scale (with respect to the ordinal nature of the data, we do not assume a normal distribution); (2) Analysis of the overall reliability of the instrument and reliability of each dimension; (3) Verification of convergent validity; (4) IRT confirmatory factor analysis with three models: six-factor correlated model, bifactor model, two-tier model which was also validated on the independent sample; (5) Mokken’s nonparametric IRT analysis to verify the scalability; (6) Analysis of DTF (i.e. the differential test functioning) across gender and relationship status; (7) In the case of absence of DTF, we made the comparison of responses of men and women as well as between singles and people in relationship through the extension of the non-parametric Mann-Whitney test for multivariate data.

## Results

### Descriptive Analysis

Descriptive statistical analysis of the items can be found in Table 1. The distribution characteristics of each item were verified testing skewness and kurtosis. Since items are ordinal, the non-normal distribution was assumed. In line with this expectation, 7 of 26 items were significantly skewed (*p* < 0.01). Furthermore, 26 of the 26 items had a significant kurtosis (*p* < 0.01). Given the results of the robust Jarque-Bera tests, all items except three were very far from normal. These results provide clear statistical evidence that the items do not have a normal distribution, and any analysis based on this assumption (Pearson correlation, principal component analysis, linear factor analysis with maximum likelihood estimation, etc.) cannot provide accurate results. This conclusion is confirmed even more by the test of multivariate normal distribution: Mardia’s test (Mardia, 1970) showed that the data do not have multivariate normal distribution (g^2^ = 602, z.kurtosis = 39, *p* < 0.001). In addition, the adjusted projection test to detect multivariate outliers (Filzmoser, Maronna, & Werner, 2008) revealed the presence of 11 of these outlying values in the data.

**Table 1:** Descriptive statistics and distributive properties of SCS items.

	M	SD	Skewness	Kurtosis	RJB	ISC

SCS-1	2.04	1.11	–0.01 ns	2.33***	1.03 ns	0.34
SCS-2	1.84	1.30	0.16**	1.89***	45.43***	0.51
SCS-3	2.54	1.11	–0.45***	2.45***	45.46***	0.41
SCS-4	1.93	1.31	0.11 ns	1.87***	40.82***	0.44
SCS-5	2.02	1.19	–0.04 ns	2.10***	15.78***	0.40
SCS-6	1.89	1.21	0.06 ns	2.03***	24.40***	0.44
SCS-7	1.96	1.34	–0.02 ns	1.81***	40.94***	0.26
SCS-8	1.86	1.18	0.14 ns	2.15***	18.70***	0.32
SCS-9	2.41	1.20	–0.32***	2.20***	45.80***	0.37
SCS-10	1.70	1.28	0.21**	1.93***	49.18***	0.30
SCS-11	2.08	1.18	–0.05 ns	2.18***	8.34*	0.41
SCS-12	1.77	1.21	0.12 ns	2.05***	27.85***	0.36
SCS-13	1.90	1.36	0.15*	1.79***	50.96***	0.52
SCS-14	2.41	1.10	–0.21**	2.31***	27.17***	0.49
SCS-15	2.21	1.19	–0.25***	2.16***	31.54***	0.53
SCS-16	1.93	1.22	0.05 ns	1.99***	29.27***	0.50
SCS-17	2.19	1.16	–0.10 ns	2.17***	16.15***	0.57
SCS-18	2.00	1.28	0.01 ns	1.91***	33.75***	0.43
SCS-19	1.79	1.13	0.11 ns	2.28***	8.76*	0.46
SCS-20	2.01	1.21	–0.05 ns	2.08***	16.10***	0.42
SCS-21	2.04	1.25	–0.02 ns	1.97***	27.12***	0.46
SCS-22	2.09	1.12	–0.05 ns	2.28***	4.28 ns	0.37
SCS-23	2.04	1.11	0.05 ns	2.24***	7.13*	0.48
SCS-24	1.95	1.23	0.06 ns	1.98***	29.75***	0.50
SCS-25	1.83	1.28	0.19**	1.93***	43.87***	0.52
SCS-26	2.12	1.07	–0.07 ns	2.38***	2.11 ns	0.51

*Note*. N = 1181. M – mean. SD – standard deviation. RJB – Robust Jarque-Bera test of normal distribution. ISC – corrected polychoric correlation of item to total score. *p < 0.05; **p < 0.01; ***p < 0.001; ns = nonsignificant.

### Analysis of Reliability

The most commonly used test of reliability is Cronbach’s α which can, however, be very inaccurate when used for ordinal scales (Dunn et al., 2014; Zumbo et al., 2007). This uncertainty can be partially corrected, when the Cronbach α is not calculated from the Pearson product-moment correlation matrix but from the polychoric correlation matrix, which takes into account the ordinal nature of the variables (Zumbo et al., 2007). Furthermore, an even better alternative is to use the McDonald ω test (Dunn et al., 2014). Hence, for the analysis of reliability we use the McDonald ω as an indicator, although for reasons of comparability we list the values of the classical Cronbach α, (calculated from the Pearson product-moment correlation matrix) and also the Cronbach α, which is calculated from the polychoric correlation matrix. Another highly desirable feature of the ω index is the possibility to validate the assumption that the instrument measures a sufficiently general construct behind all dimensions (which can be determined from the value of the hierarchical ω). Table 2 shows the values of the reliability tests for the whole range of the SCS and its individual subscales (dimensions), as well as the value of McDonald’s total and hierarchical ω. As shown in Table 2, all reliability values are relatively high. However, the value of the hierarchical McDonald ω (0.61) reveals that there is only a weak general latent factor behind the six dimensions of the SCS, which would not explain sufficient amount of the variance. Therefore, the use of a total score would be inappropriate. However, the value of the hierarchical McDonald ω for two general dimensions (0.82) means that two general factors account for 82 % of variance, so the use of two scores (13 positive and 13 negative items) should be recommended.

**Table 2:** Reliability values for SCS.

	Total	Self-compassionate dimension SK+CH+M	Self-uncompassionate dimension SJ+I+OI	SK	SJ	CH	IS	MI	OI

Cronbach *α* (Pearson)	0.86	0.84	0.86	0.78	0.68	0.69	0.74	0.68	0.71
Cronbach *α* (polychoric)	0.88	0.86	0.87	0.81	0.71	0.72	0.77	0.72	0.76
McDonald *ω*	0.92	0.90	0.89	0.81	0.72	0.73	0.78	0.73	0.74
McDonald *ω* (hierarchical)	0.61	0.82	0.82						

*Note*. SK Self-Kindness. CH Common Humanity. MI Mindfulness. SJ Self-Judgement. IS Isolation. OI Over-Identification.

### Analysis of Validity

Construct validity of the SCS was measured using Spearman’s correlations between the SCS and other instruments which measure related constructs, i.e. FSCRS, LOSC, SCCS and their respective dimensions. Correlations were in agreement to the theoretical expectations, which indicate that the SCS and its subscales show good construct validity, see Table 3.

**Table 3:** Nonparametric Spearman correlations between SCS, FSCRS, LOSC and SCCS and its subscales.

		SCS	SCS SKI	SCS SJ	SCS CH	SCS IS	SCS MI	SCS OI
	
**FSCRS**		–0.455***	–0.486***	–0.227***	–0.282***	–0.225***	–0.460***	–0.183***

FSCRS	HS	–0.303***	–0.316***	–0.202***	–0.171***	–0.171***	–0.297***	–0.118***
FSCRS	IS	–0.325***	–0.347***	–0.176***	–0.150***	–0.191***	–0.331***	–0.171***
FSCRS	RE	0.492***	0.530***	0.211***	0.369***	0.211***	0.488***	0.165***
**SCCS**		–0.113**	0.065 ns	–0.205***	0.104*	–0.196***	0.001 ns	–0.212***

SCCS	SCCS SCR	–0.437***	–0.283***	–0.473***	–0.122**	–0.326***	–0.268***	–0.418***
SCCS	SCCS SCO	0.367***	0.422***	0.272***	0.277***	0.138**	0.317***	0.219***
**LOSC**		0.291***	–0.268***	0.483***	–0.68 ns	0.529***	–0.210***	0.542***

LOSC	LOSC CSC	0.149***	–0.265***	0.360***	–0.139**	0.426***	–0.264***	0.390***
LOSC	LOSC ISC	0.366***	–0.202***	0.486***	0.027 ns	0.500***	–0.101*	0.535***

*Note*. *p < 0.05. **p < 0.01. ***p < 0.001. ns – nonsignificant. SCS – The Self-Compassion Scale (SK Self-Kindness. CH Common Humanity. MI Mindfulness. SJ Self-Judgement. IS Isolation. OI Over-Identification). FSCRS – The Forms of Self-criticism/Attacking & Self-Reassuring Scale (RE Reassured Self, IS Inadequate self, HS Hated self). LOSC – The Levels of Self-Criticism Scale (CSC Comparing Self-Criticism, ISC Internalized Self-Criticism). SCCS – The Self-Compassion and Self-Criticism Scales (SCR Self-Criticism, SCO Self-Compassion).

### IRT Factor Analysis

As already stated, there is no hope that the ordinal variables that make up the items of the questionnaire can meet the assumption of multivariate normal distribution, which is essential for the correct functioning of classical linear factor analysis (based on the maximum likelihood method of estimation). For the factor analysis, therefore, methods of IRT (item-response theory) will be used, that are much more relevant and accurate for analysing ordinal variables, given the logistic and not the linear method of their estimation.

**Figure 1:**
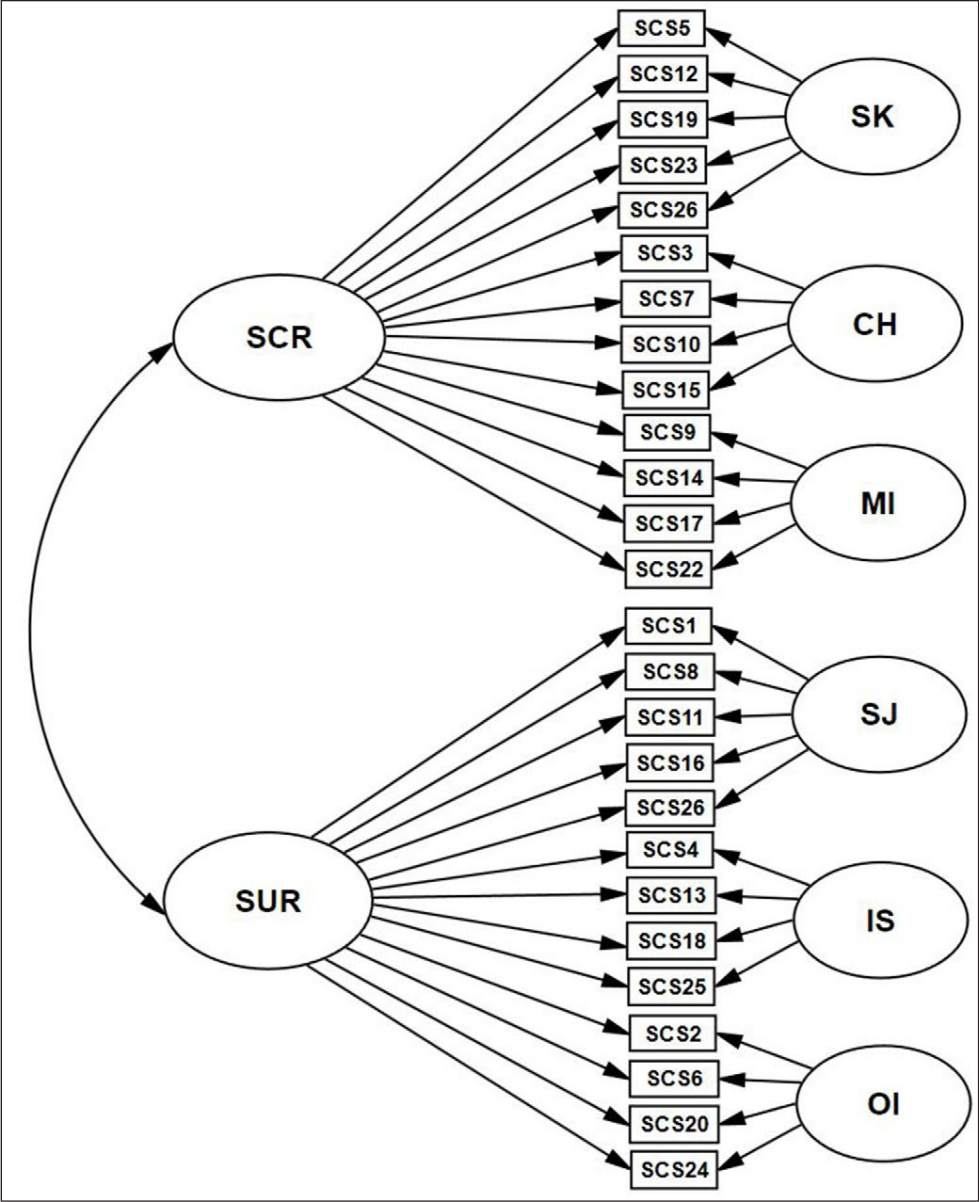
Two-tier model of the SCS scale. *Note*. SCR Self-compassionate responding subscale. SUR Self-uncompassionate responding subscale. SCS1-SCS24 particular items of SCS with numbers. SK Self-Kindness, CH Common Humanity. MI Mindfulness, SJ Self-Judgment, IS Isolation, and OI Over-identification.

The analysis will start with the confirmatory six-factor correlated IRT model, estimated in the “mirt” package (Chalmers, 2012), method of estimation is the Samejima graded response model, the algorithm is the Metropolis-Hastings Robbins-Monro algorithm which is more appropriate for highly dimensional constructs. The second model will be the bifactor IRT model (Reise, 2012; Reise et al., 2010); the method of estimation is again the Samejima graded response model. This IRT model allows testing the loadings of items for the general factor and thus estimating the proportion of variance explained by a common factor. The last model will be the two-tier IRT model (Bonifay, 2016; Cai, 2010; Cai, 2016), method of estimation is again the Samejima graded response model (see Figure 1). For verification of the fit of these models with the data we used standard indices of fit (CFI, RMSEA, SRMSR), which have their recommended thresholds CFI (>0.90 acceptable fit >0.95 excellent fit), RMSEA (<0.08 acceptable fit; <0.05 excellent fit) SRMSR (<0.08 acceptable fit; <0.05 excellent fit). For robust linear models, we also used the WRMR index which has as recommended thresholds <1.50 acceptable fit, and <1.00 excellent fit. Furthermore, we compared these models by means of the likelihood-ratio tests to determine which of them had the best absolute fit with the data. We also inspected correlations among the latent factors in the six-factor correlated model and the factor loadings of the general factor in the bifactor model to detect possible differences between positive and negative dimensions.

The 6-dimensional IRT model showed an suboptimal fit with the data (CFI = 0.80, RMSEA = 0.076, SRMSR = 0.191). Correlations within the positive (0.70, 0.62, 0.72) and negative dimensions (0.83, 0.78, 0.92) were far stronger than between positive and negative dimensions (0.28, 0.18, 0.24, 0.20, 0.22, 0.25, 0.37, 0.36, 0.42), which suggests that there are two general factors rather than a single general one. The bifactor IRT model revealed significantly better fit (CFI = 0.91, RMSEA = 0.053, SRMSR = 0.098), but a common factor did not explain a sufficient proportion of variance (hierarchical ω = 0.61), and therefore the total score can not be used. Moreover, the mean loadings for negative items (M = 0.587) are significantly higher than the mean loadings for positive items (M = 0.264) suggesting that a single general factor does not sufficiently explain the variance associated with the positive items. The two-tier model revealed the best fit (CFI = 0.95, RMSEA = 0.042, SRMSR = 0.089). Likelihood-ratio tests showed that the two-tier model has better fit than both the six-factor correlated model (χ^2^_diff_ = 795, df = 12, *p* < 0.001) and the bifactor model (χ^2^_diff_ = 972, df = 1, *p* < 0.001). In this two-tier model, the mean loadings for negative items (M = 0.589) were pretty the same than the mean loadings for the positive items (M = 0.544) suggesting that two general factors capture a sufficient amount of variance. Standardized factor loadings and explained variance of the two-tier model are shown in Table 4.

**Table 4:** Factor loadings of two-tier IRT (Samejima graded response) model.

	FG1	FG2	SK	SJ	CH	IS	MI	OI	h^2^

SCS-1	0.459	0.000	0.000	0.251	0.000	0.000	0.000	0.000	0.273
SCS-2	0.746	0.000	0.000	0.000	0.000	0.000	0.000	–0.100	0.566
SCS-3	0.000	0.534	0.000	0.000	0.203	0.000	0.000	0.000	0.326
SCS-4	0.652	0.000	0.000	0.000	0.000	0.117	0.000	0.000	0.439
SCS-5	0.000	0.509	0.541	0.000	0.000	0.000	0.000	0.000	0.551
SCS-6	0.646	0.000	0.000	0.000	0.000	0.000	0.000	–0.076	0.423
SCS-7	0.000	0.309	0.000	0.000	0.810	0.000	0.000	0.000	0.751
SCS-8	0.357	0.000	0.000	0.246	0.000	0.000	0.000	0.000	0.188
SCS-9	0.000	0.438	0.000	0.000	0.000	0.000	0.354	0.000	0.317
SCS-10	0.000	0.356	0.000	0.000	0.726	0.000	0.000	0.000	0.653
SCS-11	0.448	0.000	0.000	0.592	0.000	0.000	0.000	0.000	0.552
SCS-12	0.000	0.407	0.624	0.000	0.000	0.000	0.000	0.000	0.555
SCS-13	0.682	0.000	0.000	0.000	0.000	0.398	0.000	0.000	0.624
SCS-14	0.000	0.646	0.000	0.000	0.000	0.000	0.489	0.000	0.656
SCS-15	0.000	0.741	0.000	0.000	0.149	0.000	0.000	0.000	0.571
SCS-16	0.618	0.000	0.000	0.298	0.000	0.000	0.000	0.000	0.471
SCS-17	0.000	0.706	0.000	0.000	0.000	0.000	0.308	0.000	0.593
SCS-18	0.575	0.000	0.000	0.000	0.000	0.503	0.000	0.000	0.584
SCS-19	0.000	0.542	0.729	0.000	0.000	0.000	0.000	0.000	0.825
SCS-20	0.548	0.000	0.000	0.000	0.000	0.000	0.000	0.405	0.464
SCS-21	0.538	0.000	0.000	0.383	0.000	0.000	0.000	0.000	0.436
SCS-22	0.000	0.516	0.000	0.000	0.000	0.000	0.160	0.000	0.292
SCS-23	0.000	0.654	0.147	0.000	0.000	0.000	0.000	0.000	0.449
SCS-24	0.680	0.000	0.000	0.000	0.000	0.000	0.000	0.336	0.576
SCS-25	0.708	0.000	0.000	0.000	0.000	0.077	0.000	0.000	0.508
SCS-26	0.000	0.719	0.084	0.000	0.000	0.000	0.000	0.000	0.524

*Note*. SK Self-Kindness. CH Common Humanity. MI Mindfulness. SJ Self-Judgement. IS Isolation. OI Over-Identification.

To compare the results with a more traditional linear method of estimation, we fitted all the models with robust linear estimator WLSMV. The 6-dimensional robust linear model showed an suboptimal fit with the data (CFI = 0.88, RMSEA = 0.075, WRMR = 2.211). Correlations within positive dimensions (0.64, 0.66, 0.68) and negative dimensions (0.82, 0.77, 0.92) were far stronger than between the positive and negative dimensions (0.29, 0.18, 0.24, 0.18, 0.36, 0.19, 0.21, 0.35, 0.42). This result suggests that there are two general factors rather than a single general one. The bifactor robust linear model revealed significantly better fit (CFI = 0.93, RMSEA = 0.068, WRMR = 1.342), but a common factor does not explain a sufficient proportion of variance (hierarchical ω = 0.67), and therefore the total score can not be used. The two-tier robust linear model revealed the best fit (CFI = 0.96, RMSEA = 0.049, WRMR = 1.121). Likelihood-ratio tests showed that the two-tier model has a better fit than both the six-factor correlated model (χ^2^_diff_ = 381, df = 12, *p* < 0.001) and the bifactor model (χ^2^_diff_ = 199, df = 1, *p* < 0.001). We can therefore conclude that the more traditional linear method of estimation confirmed the results of the IRT method.

The 6-dimensional IRT model in the validation sample again showed an suboptimal fit with the data (CFI = 0.58, RMSEA = 0.116, SRMSR = 0.253). Correlations within the positive (0.77, 0.68, 0.92) and the negative dimensions (0.80, 0.78, 0.94) were far stronger than between positive and negative dimensions (0.76, 0.43, 0.46, 0.61, 0.63, 0.52, 0.46, 0.63, 0.59), which also suggests that there are two general factors rather than a single general factor. The bifactor IRT model in the validation sample revealed significantly better fit (CFI = 0.87, RMSEA = 0.066, SRMSR = 0.080), but a common factor again does not explain a sufficient proportion of variance (hierarchical ω = 0.68), and therefore the total score can not be used. The two-tier model again revealed the best fit (CFI = 0.93, RMSEA = 0.056, SRMSR = 0.071). Likelihood-ratio tests show that the two-tier model has a better fit than both the six-factor correlated model (χ^2^_diff_ = 488, df = 12, *p* < 0.001) and the bifactor model (χ^2^_diff_ = 333, df = 1, *p* < 0.001).

### Mokken’s Analysis of Scalability

Mokken’s analysis (Sijtsma & Molenaar, 2002) allows checking whether the items are scalable into a single scale. It uses covariances between pairs of items to test the monotonic model – if a model satisfies the test of scalability and monotonicity, it is safe to use the total score: items are scalable into a single scale. Unlike the Rasch’s model, it does not assume any parametric shape of function response of items, so it is a nonparametric IRT model. The Self-compassionate responding scale (SK + CH + MI) is scalable after discarding of items 9 and 22, its coefficient H = 0.339 (SE = 0.013), which is above the recommended threshold 0.300. The Self-uncompassionate responding scale (SJ+IS +OI) is scalable after the discarding of item 8, value of coefficient H = 0.348 (SE = 0.012). Obviously, it makes no sense to sum up the total score of the whole questionnaire of the SCS (26 items), since its scalability is very low (H = 0.207, SE = 0.009).

### Analysis of Differential Test Functioning

Concerning the invariance of the test to different demographic groups, differential item functioning (DIF) is the most commonly used analysis in the context of IRT. However, it is more appropriate to verify differential test functioning (DTF) (Chalmers et al., 2016): Recent research showed that even if one or several items displayed a significant DIF, this does not necessarily imply that the test would display the DTF as a whole, for it might happen that the DIF of one item (i.e. a different probability of responses of members of one group as compared to members of the second group, while the value of the latent ability is the same) is compensated by another item. We are particularly interested in the DTF across latent ability (signed DTF), because this may create systematic distortion of the total score at the disadvantage of one group (for the sake of completeness, let us also add that the DTF for a particular part of the latent ability can be verified – unsigned DTF).

Concerning gender, values of the signed DTF (i.e. the average distorted score, which is in this case in the advantage/disadvantage of men) are 0.18 for the subscale Self-compassionate responding (SK + CH + MI), which is 0.34% and represents a non-significant difference (*p* = 0.65), and –0.05 for the subscale Self-uncompassionate responding (SJ + IS + OI), which is –0.10%, and also represents a non-significant difference (*p* = 0.88). In the case of the difference between the singles and people in relationship, values of the DTF (the advantage/disadvantage of singles) are –0.47 for the subscale Self-compassionate responding (SK +CH+M), which is –0.90% and represents a non-significant difference (*p* = 0.18), and 0.35 for the subscale Self-uncompassionate responding (SJ + IS + OI), which is 0.68%, and also represents a non-significant difference (*p* = 0.27).

Note that these values represent a systematic distortion of the test and they cannot be confused with the differences in the total scores or latent ability scores between the groups. Invariance of the test is just a prerequisite for an accurate comparison of groups and is a real, although unfortunately extremely widespread problem of how to perform the comparison of groups (e.g., t-test, Mann-Whitney nonparametric test, etc.) without ascertaining whether the scores, which are to be compared, are or are not systematically distorted in the advantage and disadvantage of any of the groups.

Because the DTF did not show any significant systematic distortion, we can test possible differences in scoring responses. Due to the multivariate non-normal distribution and the presence of outliers we use an extension of (projection type) the non-parametric Mann-Whitney test for multivariate data (Wilcox, 2005). In this test, it is verified if there is a probability of ranking significantly deflected for one of the groups – therefore the null hypothesis is that the value of η is 0.5 (i.e. between the groups there is no difference) and the test verifies if the estimate of this value is significantly different from 0.5. The testing of the responses between gender resulted in an estimated value of η = 0.51 which is obviously a very insignificant amount (95% CI 0.23 – 0.80). The same goes for testing differences in the responses between singles and people in relationship, which reveals an estimated value of η = 0.56 (95% CI 0.14 – 0.84). In conclusion, we can say that there is no difference in the responses between men and women and between people in relationship and single people.

### Development of Norms

Because all subscales are scalable, and the total score is invariant in respect to its items, we also provide here the norms calculated for the total score in each subscale, i.e., for the subscales of Self-compassionate responding and Self-uncompassionate responding (Table 5). Based on Mokken’s analysis (see above), however, we had to exclude three items: 9 and 22 in the first scale, and 8 in the second scale. Thus, the first scale (Self-compassionate responding) contains 11 items, and the range of scores is from 11 to 55, and the second scale (Self-uncompassionate responding) contains 12 items, and its range score is between 12 and 60. These norms can serve to approximately provide the differentiation degree of Self-compassionate responding and Self-uncompassionate responding within a selected population.

**Table 5:** Percentil (rank) norms for subscales.

Self-compassionate responding SK+CH+MI		Self-uncompassionate responding SJ+IS+OI	

Score	Percentile rank (%)	Score	Percentile rank (%)

11	0.3	12	0.3
12	0.3	13	0.3
13	0.3	14	0.4
14	0.4	15	0.8
15	0.8	16	0.9
16	0.9	17	1.3
17	1.3	18	1.9
18	1.9	19	2.5
19	2.5	20	4.1
20	4.1	21	5.3
21	5.3	22	7.8
22	7.8	23	10.2
23	10.2	24	12.6
24	12.6	25	15.8
25	15.8	26	19.2
26	19.2	27	22.7
27	22.7	28	26.7
28	26.7	29	31.2
29	31.2	30	36.2
30	36.2	31	40.5
31	40.5	32	44.9
32	44.9	33	49.7
33	49.7	34	54.2
34	54.2	35	59.1
35	59.1	36	63.6
36	63.6	37	68.0
37	68.0	38	72.1
38	72.1	39	76.3
39	76.3	40	78.9
40	78.9	41	82.6
41	82.6	42	85.1
42	85.1	43	87.9
43	87.9	44	90.2
44	90.2	45	92.4
45	92.4	46	94.5
46	94.5	47	95.9
47	95.9	48	97.1
48	97.1	49	97.7
49	97.7	50	98.1
50	98.1	51	98.9
51	98.9	52	99.3
52	99.3	53	99.4
53	99.4	54	99.7
54	99.7	55	100.0
55	100.0	56	100.0
		57	100.0
		58	100.0
		59	100.0
		60	100.0

*Note*. SK Self-Kindness. CH Common Humanity. MI Mindfulness. SJ Self-Judgement. IS Isolation. OI Over-Identification.

## Discussion

The aim of this paper was to translate The Self-Compassion Scale (SCS Neff, 2003) into Slovak language and to verify its psychometric properties and the factor structure through item response theory (IRT). The primary reason for the translation of this scale is that no measure of self-compassion in Slovak language is currently available. Through validating the translated SCS, which is the most widely used measure of self-compassion currently available, we enable future research on self-compassion among Slovak populations. Also, we were interested in verifying either the original factor structure reported by Neff (2003) that included six subscales and one higher-order ‘self-compassion’ factor, or a two general factors solution reported by other authors (e.g. Brenner et al., 2017; Brown et al., 2016; Costa et al., 2016; López et al., 2015; Muris & Petrocchi, 2016). Finally, we also tested the reliability, validity of this scale and created norms, to enable use of the Slovak translation of the SCS in future research.

We found evidence of very good internal consistency for the total score (0.86) and a good internal consistency for each dimension (between 0.68 and 0.78) as measured by Cronbach’s alpha. These reliability coefficients are slightly lower, but comparable to the reliability coefficients reported in the original validation study (0.92 for the whole scale and for the various dimensions it varied between 0.75–0.81) (Neff, 2003). Likewise, other studies of both English and translated versions of the SCS have reported comparable reliability coefficients, demonstrating that the SCS is a consistent measure of self-compassion across a range of languages (e.g. Benda & Reichova, 2016; Deinz et al., 2008; Petrocchi et al., 2014; Van Dam et al., 2011).

In the current study, we found that a two-tier model fitted the present data, thereby confirming that one common ‘self-compassion’ factor does not explain a sufficient proportion of the total scale variance to justify using an aggregated score for the entire scale. In contrast, the scale is better divided into two subscales: Self-uncompassionate responding composed of the dimensions Self-Judgement, Isolation, Over-identification; and Self-compassionate responding consisting of the dimensions of Mindfulness, Common humanity and Self- Kindness. Therefore, our findings about the factor structure of the Slovak version of SCS support a growing body of research recommending the use of two factors (positive and negative self-compassion) for the SCS (Brenner, Heath, Vogel, & Credé, 2017; Brown, Bryant, Brown, Bei & Judd, 2016; Costa, Marôco, Pinto-Gouveia, Ferreira, & Castilho, 2016; López, Sanderman, Smink, Zhang, van Sonderen, Ranchor, & Schroevers, 2015; Muris & Petrocchi, 2016).

The results of confirmatory factor analyses of previous studies (Benda, & Reichová, 2016; Neff, 2003; Petrocchi et al., 2014) are very difficult to compare with our results because earlier research utilised less than suitable estimation methods (e.g. the method of maximum likelihood) for ordinal items which are exhibiting, as the authors themselves admit, a significantly non-normal distribution. Improperly used estimation methods may lead to incorrect values of fit indices as well as to wrong estimations of parameters (Benda, & Reichová, 2016; Deniz et al., 2008; Neff, 2003; Petrocchi et al., 2014; Van Dam et al., 2011). For example, in the Czech study (Benda, & Reichová, 2016) the authors used the method maximum likelihood (ML, which is the default estimation method in AMOS 23) for the estimation of the parameters, which in the case of a multivariate non-normal division misrepresents indices of fit as well as the estimation of parameters. Moreover, the original six-factor model does not have acceptable values of indices of fit, and the authors solved this problem by omitting 6 items and then repeated the confirmatory analysis with the same set of data. This is a procedure that psychometric literature does not recommend (Brown, 2006). Bearing in mind that the size of their sample was quite impressive (N = 5,638), the authors might have better used a standard validation procedure, that is, to perform the first step of analysis on the first half of the sample, and then to verify the modified instrument with the second half of the sample. Also, in the original study (Neff, 2003), the author worked with the same set of data in an exploratory factor analysis and a confirmatory factor analysis, and the estimation method was not mentioned, but it is probably the ML as well. Finally, in another study (Petrocchi et al., 2014), adequate model fit was obtained through excluding two problematic items, and again authors used the ML method of estimation. Multidimensional IRT models are much more suitable for the analysis of ordinal data and enable far more accurate estimations of parameters (Chalmers, 2012).

The verification of construct validity in previous studies proceeded with quite distant constructs such as self-esteem and self-acceptance (Benda & Reichová, 2016), self-esteem and satisfaction with life (Deniz et al., 2008), and social connectedness at work (Neff, 2003), and because of this, it is hard to compare their results with our results. In our study, we consistently used the same or closely related constructs, i.e. self-criticism, self-compassion and and self-reassurance, which was also possible due to the fact that in the meantime several studies came out with new scales to measure these constructs. The only comparison possible is the correlation of the SCS Self-compassion scale with the Self-Criticism subscale from The Depression Experience Questionnaire (Blatt, D’Afflitti, & Quinlan, 1979), r = –0.65, *p* < 0.01, reported by Neff (2003). Likewise, we obtained relatively high Spearman’s correlation coefficients between the SCS and the more recent scales we included for measuring self-criticism and self-compassion, i.e. FSCRS, SCCS and LOSC. We used Spearman’s correlations due to the non-normal distribution of the total scores and the presence of outliers. Given that the authors (Benda, & Reichová, 2016; Neff, 2003; Petrocchi et al., 2014) do not mention the scale properties of the total score; it is questionable whether the use of Pearson’s correlation was appropriate.

A benefit of the present study is the convergent validation with a relatively new scale SCCS (Falconer, King, & Brewin, 2015), which also showed significant correlation coefficients in accordance with the theoretical assumptions. Thus in our study, the construct validity of SCS was investigated with many other existing scales that primarily measure self-criticism, self-compassion or self-reassurance.

Given the size of the sample (1181 respondents for the research sample and 676 respondents for the independent sample), we were able to use the models of item response theory (IRT). IRT analysis showed that the two major dimensions Self-compassionate responding and Self-uncompassionate responding meet the conditions of a good fit with data, that their general factors explain a sufficient proportion of the variance, and that the psychometric properties of individual items are affordable as well. We also found that the test is invariant, meaning that it can be applied to both genders (men and women), and also in the context of relationship status (single people and people in relationship).

Despite the size of our sample and our independent validation sample, the sample is not representative of the Slovak population, especially regarding age and education, which limits the study and could lead to low external validity. Therefore, the results could not be simply generalized to the whole Slovak population and further research is needed.

The practical benefit of this article is the development of norms for the two main subscales of the SCS, Self-compassionate responding and Self-uncompassionate responding, with which we can diagnose and discern highly self-compassionate and highly self-uncompassionate people from the general population. Norms were created, but considering the non-representation, they do not represent the distributions of Self-uncompassionate responding and Self-compassionate responding in the population. In future research the samples studied should be extended to a clinical population and various diagnoses, so that it is possible to distinguish people with the pathological Self-uncompassionate responding from people with high Self-uncompassionate responding within normality.

## Conclusion

The Self-Compassion Scale is a reliable and valid instrument to measure the level of Self-compassionate responding and Self-uncompassionate responding in the Slovak language version. In the future, it would be beneficial to check its use in a clinical setting, and to construct norms using a large representative sample of the Slovak population.

## Additional Files

The additional files for this article can be found as follows:

10.5334/pb.398.s1 Appendix 1 The Self-compassion scale (Neff, 2003) – items of English and Slovak versions.
