# Supplementary material for: Self-Compassion Scale: IRT Psychometric Analysis, Validation, and Factor Structure – Slovak Translation
Source: Psychol Belg. 2018 Jan 4;57(4):190–209. doi: 10.5334/pb.398 (PMC6194536; doi:10.5334/pb.398)
Supplement: Appendix 1 — The Self-compassion scale (Neff, 2003) – items of English and Slovak versions. [file pb-57-4-398-s1.pdf]

Appendix 1

The Self-compassion scale (Neff, 2003) – items of English and Slovak versions

1. I'm disapproving and judgmental about my own flaws and inadequacies. SJ
   Som odmietavý/á a posudzovačný/á voči svojim vlastným chybám a nedostatkom. SJ
2. When I'm feeling down I tend to obsess and fixate on everything that's wrong. OI
   Keď som nešťastný/á, zvyknem byť posadnutý/á myšlienkami na všetko, čo v mojom živote nie je v poriadku. OI
3. When things are going badly for me, I see the difficulties as part of life that everyone goes through. CH
   Keď mi veci nevychádzajú, ťažkosti vnímam ako súčasť života, ktorou prechádza každý človek. CH
4. When I think about my inadequacies, it tends to make me feel more separate and cut off from the rest of the world. IS
   Keď rozmýšľam nad svojimi nedostatkami, zvyknem sa cítiť iný/á a odrezaný/á od zvyšku sveta. IS
5. I try to be loving towards myself when I'm feeling emotional pain. SK
   Keď cítim emocionálnu bolesť, snažím sa byť k sebe láskavý/á. SK
6. When I fail at something important to me I become consumed by feelings of inadequacy. OI
   Keď neuspejem v niečom, čo je pre mňa dôležité, som pohltený/á pocitmi vlastnej nedostatočnosti. OI
7. When I'm down and out, I remind myself that there are lots of other people in the world feeling like I am. CH
   Keď som psychicky na dne, pripomínam si, že na svete je mnoho ďalších ľudí, ktorí sa cítia rovnako ako ja. CH
8. When times are really difficult, I tend to be tough on myself. SJ
   V ťažkých časoch mám tendenciu byť sám/sama na seba tvrdý/á. SJ
9. When something upsets me I try to keep my emotions in balance. MI
   Keď ma niečo rozruší, snažím sa udržať svoje emócie v rovnováhe. MI
10. When I feel inadequate in some way, I try to remind myself that feelings of inadequacy are shared by most people. CH
    Keď sa v niečom cítim nedostatočný/á, pripomínam si, že takéto pocity nedostatočnosti má väčšina ľudí. CH
11. I'm intolerant and impatient towards those aspects of my personality I don't like. SJ
    Nemám pochopenie ani trpezlivosť voči aspektom svojej osobnosti, ktoré sa mi nepáčia. SJ
12. When I'm going through a very hard time, I give myself the caring and tenderness I need. SK
    Keď mám veľmi ťažké obdobie, zahŕňam sa súcitom a nehou, ktoré potrebujem. SK
13. When I'm feeling down, I tend to feel like most other people are probably happier than I am. IS
    Keď som na dne, zvyknem mať pocit, že väčšina ľudí je pravdepodobne šťastnejšia ako ja. IS
14. When something painful happens I try to take a balanced view of the situation. MI
    Keď sa mi prihodí niečo bolestivé, snažím sa na danú situáciu pozerať vyvážene. MI
15. I try to see my failings as part of the human condition. CH
    Svoje zlyhania sa snažím vnímať ako prejavy ľudskosti. CH
16. When I see aspects of myself that I don't like, I get down on myself. SJ
    Keď na sebe pozorujem časti seba, ktoré sa mi nepáčia, neustále sa kritizujem. SJ

17. When I fail at something important to me I try to keep things in perspective. MI
    Keď zlyhám v niečom, čo je pre mňa dôležité, snažím sa danú situáciu vnímať s nadhľadom. MI
18. When I'm really struggling, I tend to feel like other people must be having an easier time of it. IS
    Keď s niečím naozaj bojujem, zvyčajne cítim, že to ostatným ľuďom musí ísť ľahšie. IS
19. I'm kind to myself when I'm experiencing suffering. SK
    Keď trpím, som k sebe láskavý/á. SK
20. When something upsets me I get carried away with my feelings. OI
    Keď ma niečo rozruší, strácam kontrolu nad svojimi pocitmi. OI
21. I can be a bit cold-hearted towards myself when I'm experiencing suffering. SJ
    Keď trpím, viem byť k sebe trochu bezcitný/á. SJ
22. When I'm feeling down I try to approach my feelings with curiosity and openness. MI
    Keď sa cítim zle, snažím sa ku svojim pocitom pristupovať so zvedavosťou a otvorenosťou. MI
23. I'm tolerant of my own flaws and inadequacies. SK
    Som tolerantný/á voči vlastným chybám a nedostatkom. SK
24. When something painful happens I tend to blow the incident out of proportion. OI
    Keď sa mi prihodí niečo bolestivé, zvyknem danú udalosť vnímať horšie než aká v skutočnosti je. OI
25. When I fail at something that's important to me, I tend to feel alone in my failure. IS
    Keď zlyhám v niečom, čo je pre mňa dôležité, zvyknem sa v mojom neúspechu cítiť osamelo. IS
26. I try to be understanding and patient towards those aspects of my personality I don't like. SK
    Snažím sa byť chápavý/á a trpezlivý/á voči tým častiam mojej osobnosti, ktoré sa mi nepáčia. SK

Note. SK Self-Kindness, CH Common Humanity. MI Mindfulness, SJ Self-Judgment, IS Isolation, and OI Over-identification.
